# A Novel Online Dissection Course on Lower Limb Anatomy During the COVID-19 Pandemic

**DOI:** 10.7759/cureus.23081

**Published:** 2022-03-11

**Authors:** Sunit V Jadhav, Vaishaly K Bharambe, Varun S Pathak, Ananya P Khurjekar, Raghav L Navandar, Arunprasad V K.

**Affiliations:** 1 Anatomy, Symbiosis Medical College for Women, Pune, IND; 2 Anatomy, Byramjee Jeejeebhoy Government Medical College and Sassoon General Hospital, Pune, IND; 3 Medicine, Byramjee Jeejeebhoy Government Medical College and Sassoon General Hospital, Pune, IND

**Keywords:** modern medical teaching methods, covid-19, distance learning, medical education, e-learning, student feedback, anatomy education, online dissection, online education

## Abstract

Introduction: The teaching of human anatomy, a medical subject that relies heavily on live teaching, teacher-student interactivity, and visuospatial skills, has suffered tremendously since the COVID-19 pandemic mandated the shutting down of medical institutions. The medical education fraternity was compelled to replace the traditional teaching method of hands-on cadaveric dissections (HOCDs) with online education to overcome this new challenge, but it came at the cost of reduced student engagement and lesser spatial orientation.

Method: In this cross-sectional, questionnaire-based study, we designed a novel online dissection course on lower limb anatomy and collected student feedback on the same from consenting Phase I Bachelor of Medicine, Bachelor of Surgery (MBBS) students of Symbiosis Medical College for Women, Pune, India. The course design consisted of three different modes: a live Zoom session using a handheld camera phone, a pre-recorded video dissection uploaded on the institute learning management system, and a Powerpoint presentation with high-resolution photographs of each dissected layer; and the feedback intended to find out what works best for the students. Overall feedback regarding their preferences in terms of presentation design, use of background music in pre-recorded videos, and overall learning experience was also collected. The course consisted of six two-hour teaching sessions. The first three sessions each used a different mode of teaching, repeating the same pattern in the next three sessions. The first mode of teaching implemented was a live Zoom session where instructors used a hand-held cell phone camera to show specimens that had been dissected a day prior. The second mode involved a pre-recorded video showing step-by-step dissection performed by the instructor which was then uploaded on the Institute Learning Management System. Of the two pre-recorded videos, background music consisting of a low-volume instrumental track was added to the second video. The third mode utilized Powerpoint presentations containing high-resolution photographs of each dissected layer on a separate slide along with labeling. The presentations were shown to the students over a Zoom call. A Google Form (GF) questionnaire was created after validation by subject experts to gather the students’ feedback on the teaching and learning of anatomy via these sessions. The GF responses were collected and analyzed using Microsoft Excel.

Results: 41.7% of students recommended the use of a combination of all three modes in the same session, while 36.7% favored pre-recorded videos. 86.7% of students said that a good quality presentation design helps in keeping them engaged and only 23% of students favored the use of background music for increasing their ability to concentrate. 63.3% of students found the learning experience highly satisfactory.

Conclusion: Although virtual dissection teaching methods may not be able to completely replace HOCDs, a well-planned online dissection course incorporating multiple modes of online dissections with an emphasis on good quality presentation design and frequent teacher-student interactivity can provide a strong impetus for learning in the absence of live teaching methods.

## Introduction

Human anatomy is one of the basic sciences that is taught in the medical curriculum worldwide [1,2]. For many years this subject has been taught using traditional teaching methods such as didactic lectures, demonstrations, small group discussions, and cadaveric dissections. With pedagogical evolution, anatomical education started witnessing the ingraining of newer, creative teaching methods which incorporated computer-assisted learning [3]. Online educational content delivered through websites such as YouTube has been shown to augment students’ learning experiences [4]. The inclination towards such teaching tools was further accentuated after restrictions on the live classroom teaching setup due to the 2020 COVID-19 pandemic [5]. Distance education became the new norm and replacing old school methods with newer modalities like streaming video platforms, web conferencing tools, and much recently, virtual anatomy atlas software, was no longer just an evolutionary trend but an absolute necessity [6,7]. Furthermore, the incorporation of learning management systems such as Google Classroom [8] and Moodle [9,10] in medical education has made it possible to facilitate the teaching and learning of this complex subject in an orderly manner, in spite of the physical distance. Two important drawbacks are to be considered in this new wave of teaching, however: the first being the lack of live face-to-face interaction [11,12], the second a deprivation of visuospatial learning that is a hallmark feature of anatomy learning, especially in hands-on cadaveric dissections (HOCDs) [13]. The understanding of visual and spatial relationships amongst structures offered by HOCDs has always made this method an integral, irreplaceable part of anatomy teaching and learning [14]. The inability to conduct such sessions during institutional lockdowns is a matter of concern for anatomy education [15,16]. Students’ perspectives on the very same matter have been studied in recent times [17,18]. In March 2020, Diaz et al. ran an online anatomy practical course consisting of 20 pre-recorded videos of prosected cadaveric specimens with a positive response and statistically significant rise in academic scores [19]. Since then, various other methods have been used such as virtual dissection tables, dissection audio-visual resources (DAVR), and dissection educational videos (DEVs) [20-24]. In spite of multiple studies focussing on student feedback on online anatomy education, no studies have collected feedback by comparing multiple modes of online dissection within the same course. Whether the “touch-and-feel” based learning of HOCDs can ever be replaced by virtual dissections remains an issue of debate, but an effort to keep an online dissection session as real as possible can be done in today’s times, thanks to the availability of enhanced digital audio-video creation technology. In this study, the authors have attempted to understand students’ perspectives on a novel online lower limb dissection course that they designed and delivered during the pandemic. The course encompasses various possible modes of online dissection teaching. Factors such as preferred mode of lecture delivery, presentation design, the scope for background music in online sessions, and overall learning experience have been considered.

## Materials and methods

This was a questionnaire-based study exploring students’ perspectives on a novel online dissection course on lower limb anatomy. The course was a part of the online anatomy curriculum of a Phase I MBBS batch at Symbiosis Medical College for Women, Pune. It was mentioned in the student consent form that the feedback will be collected anonymously for purposes of medical education research and curricular development. Sixty students gave consent and were included in the study. Before collecting the data, the study plan was proposed to the Institute Research Committee at Symbiosis Medical College for Women and the approval number received was SMCW/IRC/Fac Res/52/2022. As per Table 4.2, Serial Number 1 of the National Ethical Guidelines for Biomedical and Health Research involving Human Participants of the Indian Council of Medical Research, New Delhi, studies involving comparison of instructional techniques, curricula, or classroom management methods are exempted from Ethical Committee review.

Course design

The course consisted of six two-hour teaching sessions, conducted for the entire batch. Three sessions were conducted initially, each one using a different mode of teaching (Tables 1, 2). The same pattern was repeated for the next three sessions.

**Table 1:** Modes of Online Dissection Teaching Sessions and Materials Used

No.	Mode of Teaching	Material Used
1	LIVE Zoom Session by using the hand-held cell phone camera to show prosected specimen (LIVE ZS)	Xiaomi Redmi Note 9 Pro Max Mobile Phone
2	Pre-recorded video showing step-by-step dissection uploaded on the Institute Learning Management System (PR Video)	DJI Osmo Pocket Handheld 12 MP camera, LumaFusion for iPad Moodle Version 3.9
3	PowerPoint presentation with high-resolution photographs of each dissected layer (PPT)	Canon DSLR PowerShot SX70 HS camera, Microsoft PowerPoint by Microsoft Office Professional Plus 2016

**Table 2:** Course Design LIVE ZS: Live Zoom session; PR Video: Pre-recorded video; PPT: PowerPoint presentation

No.	Topic of Session	Mode of Teaching
1	Thigh - Anterior Compartment	LIVE ZS
2	Thigh - Medial Compartment	PR Video
3	Thigh - Posterior Compartment	PPT
4	Leg - Anterior and Lateral Compartment	LIVE ZS
5	Leg - Posterior Compartment	PR Video
6	Foot	PPT

Session design

Live Zoom Session Over the Cellphone

In this mode, the dissection was done a day prior by the instructor. The prosected specimen was then shown over a live Zoom session by two teachers. One teacher held the cellphone (Redmi Note 9 Pro Max; Xiaomi, Beijing, China) whose camera was used to show the specimen while the other teacher taught with the use of forceps for pointing and grasping the necessary structures. Students had the liberty to interrupt the teacher in the middle of the session by unmuting themselves and asking questions, whenever needed. The total duration of the session was two hours.

Pre-recorded Videos Uploaded on Institute’s Learning Management System

In this mode, the dissection was done a day prior by the instructor. The entire video was first recorded using the high-definition DJI Osmo Pocket Handheld 12 MP camera (DJI, Shenzhen, China). The video consisted of a moving camera format with close-ups on certain structures as well as a fixed camera format when the concerned regional anatomy was summarized. The video was then edited using LumaFusion for iPad and exported with the specifications of 1920 x 1080 pixels at 30 frames per second. During the editing, while the parts of the video wherein the instructor is teaching were kept at normal speed, the parts of the video where the instructor is dissecting in real-time were sped up significantly. This way, the unnecessary length of dissection time was shortened, yet various layers of the dissection were demonstrated step-by-step to the viewers. Of the two videos (Table 2, Numbers 2 and 5), each of 40 minutes duration, that were produced with this method, an instrumental track in low volume was added as background music in the second video. Both of these videos were further divided into two halves of 20 minutes duration. Thus, two 20 minute videos were shown to the students in each session. Both the sessions started with the instructor navigating the students through the LMS in a 10-minute online briefing over Zoom call. The students were then given 30 minutes to watch the first part of the video on their respective devices. This was followed by a 30-minute live interactive Question and Answer (Q&A) session over Zoom call. The students then watched the second part of the video over the next 30 minutes, again on their respective devices, followed by yet another live question and answer session over the same Zoom call. The total duration of the session was two hours.

PowerPoint Presentation

In this mode, dissection was done a day prior by the instructor, and high-resolution photographs were taken using a Canon DSLR PowerShot SX70 HS camera (Canon, Tokyo, Japan). The presentation consisted of a step-by-step dissection with the photograph of each dissected layer on a separate slide along with labeling. The presentation was screen-shared live over the videoconferencing application Zoom. Students had the liberty to interrupt the teacher in the middle of the session by unmuting themselves and asking questions, whenever needed. The total duration of the session was two hours.

Questionnaire design

A Google Form (GF) questionnaire was created to gather the students’ feedback on the teaching and learning of anatomy in these sessions. It had three sections. The first section consisted of consent. It was mentioned here that the feedback will be used anonymously for purposes of research and improved delivery of online anatomical education in the future. The second section consisted of feedback on lower limb dissection sessions in relation to student preferences on the mode of teaching, the effect of presentation design and annotations on students’ ability to stay focussed, and students' perception of the use of background music in pre-recorded dissection videos. The third section collected overall feedback on the session regarding parameters such as speed of lecture, degree of repetition of information, level of teacher-student interaction in the session, comprehensibility of the topic taught, and overall quality of the students’ learning experience. After thorough validation by subject experts, this questionnaire was distributed to Phase I MBBS students of Symbiosis Medical College for Women, Pune. The students not willing to participate in the study were given the opportunity to withdraw from the study.

Data analysis

The GF responses were collected and analyzed using Microsoft Excel. An ascending Likert scale was used to assess students' comprehension of the taught topics and overall learning experience. This data was visually represented as bar charts. Pie charts were used to represent the student responses on preferred session mode, perception of design and annotation use in PowerPoint presentations, and student views on the use of background music in pre-recorded videos.

## Results

**Figure 1:**
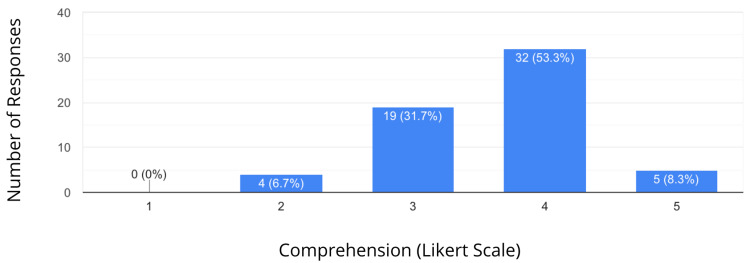
Comprehension of the Topic

**Figure 2:**
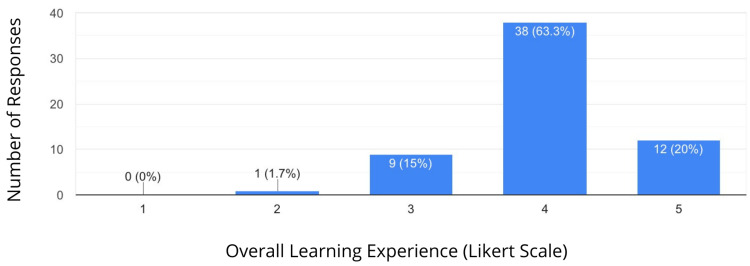
Overall Learning Experience

The results showed that 53.3% of students rated comprehension of the topics taught as a 4 on a 5-point ascending Likert scale. Similarly, 63.3% of students found the overall learning experience highly satisfactory (Figures 1, 2).

**Figure 3:**
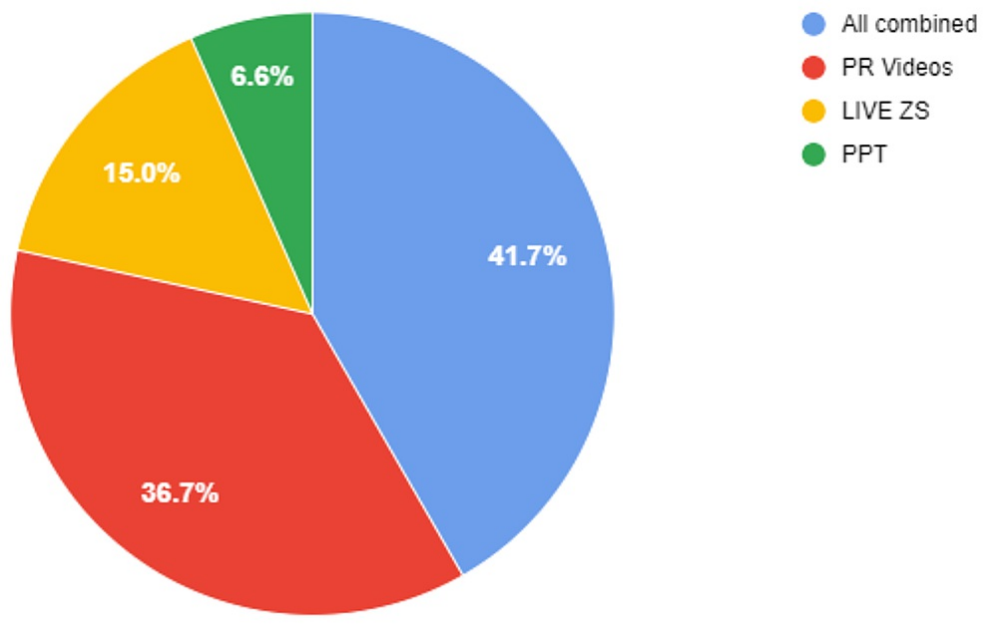
Preferred Mode of Session LIVE ZS: Live Zoom session; PR Video: Pre-recorded video; PPT: PowerPoint presentation

In terms of mode of lecture delivery, 41.7% of students recommended that the instructor use a combination of all three modes in the same lecture. 36.7% of students preferred the PR videos, 15% preferred the live ZS, and 6.6% preferred a PPT (Figure 3).

**Figure 4:**
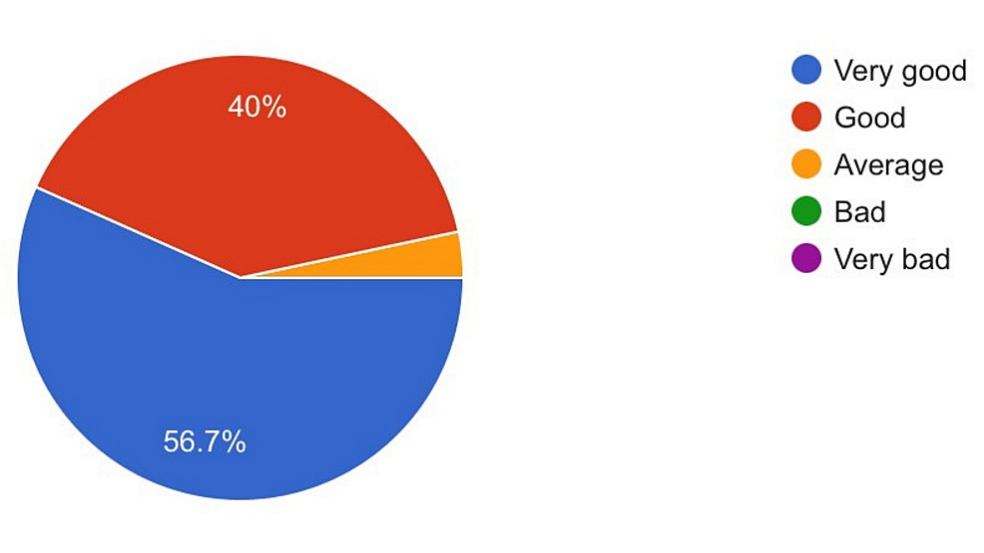
Design of the Presentation

**Figure 5:**
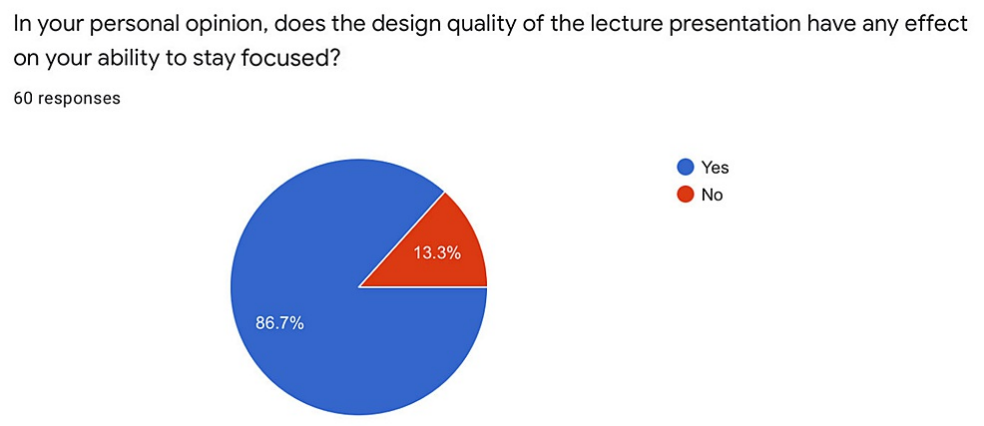
Effect of Presentation Design on students' focus

**Figure 6:**
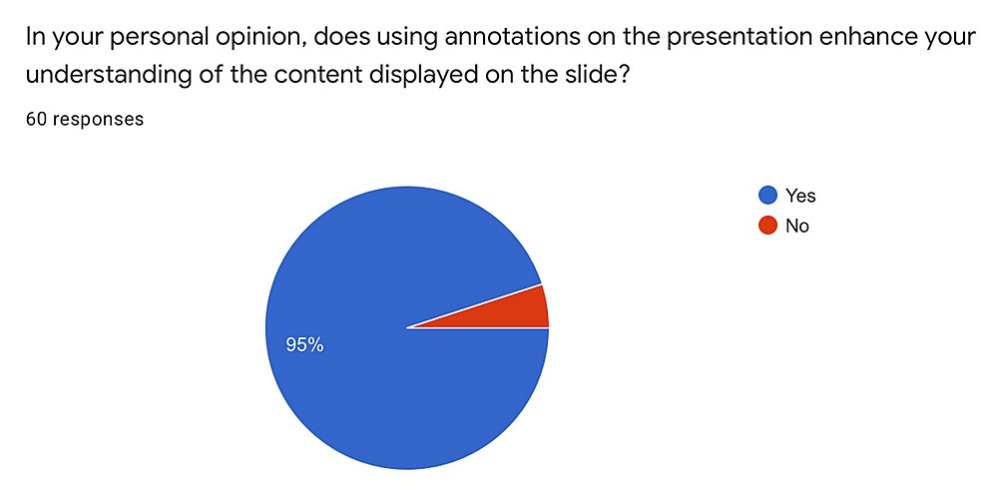
Effect of Annotations

56.7% of participants rated the PowerPoint presentation design as a 4 on a 5-point ascending Likert scale. 86.7% of students opined that the quality of presentation design directly correlates with their ability to stay focused during the lecture. 95% of students said that the annotations helped enhance their understanding of the presentation content (Figures 4, 5, 6).

**Figure 7:**
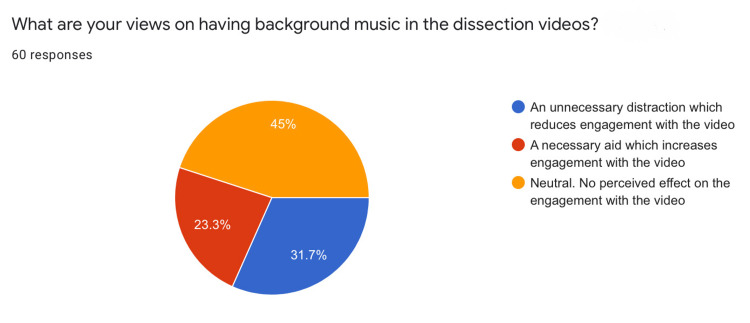
Effect of Background Music

Out of two PR videos used in the study, the background music was added to the second one. The research attempted to assess how this affected student engagement (Figure 7).

Forty-five percent of students were indifferent to the usage of background music in the dissection videos and perceived no effect on their engagement with the video. 23.3% thought that the music increased engagement, whereas 31.7% considered it as an unnecessary distraction.

## Discussion

The present study aimed at understanding student preferences in online dissection sessions on lower limb anatomy with the objective of improving curricular design for future online learning sessions based on the feedback collected. The course incorporated three modes of online dissections: live Zoom sessions, pre-recorded videos of dissection, and PowerPoint presentations of the dissected specimens. 

Preferred mode of lecture delivery

Most students suggested that the instructor incorporate a combination of all modes in the same online session (Figure 3). However, if they had to choose one, 36.7% of students favored pre-recorded videos. In a similar study by Choi-Lundberg et al. on the effectiveness of using DAVR as a preliminary mode of learning dissection, the authors reported that implementing DAVR resulted in higher scores among undergraduate medical students. A positive correlation was noted between scores and the number and total time of DAVR viewed [22]. PPTs were the least preferred modes of teaching online dissection. The following factors could be responsible for greater student preference for PR videos:

Control of Video Playback

Students could pause, replay, speed up or slow down the pre-recorded videos on their own devices. This gave them the liberty to focus on different parts of the dissection as per their understanding. The live videos had the teacher progressing at a fixed pace, with no option to pause or replay. The PPTs had static images which might have failed to capture visuospatial intricacies, leading to low preference. Fidalgo et al. conducted a similar study to assess the use of alternative learning methods among students in which students clearly preferred a video format over PPTs. However, they concluded that the most beneficial way to aid most students’ learning would be to administer a combination of different alternative teaching methods [25].

Elimination of Internet Bandwidth Issues

A major drawback of the live ZS could be the reduced video quality, audio delay, and buffering experienced due to server overload. These problems do not arise while viewing pre-recorded content. In a similar study by John et al. [26], nearly 60% of students preferred PR videos over live videos due to this very reason.

Reduced Dissection Time

The parts of the PR video where the teacher was dissecting in real-time were sped up in order to reduce the length of dissection time. However, while demonstrating the various layers and structures, the video speed was brought back to normal. Not only was this the closest imitation of an actual HOCD, but it also allowed the students to focus on important anatomical structures better.

More Teacher-Student Interaction

Two slots of 30 minutes each were allotted purely for interactive Q&A sessions over Zoom call after watching the first and second parts of the PR videos. In the live ZS as well as the PPT modes, students had to unmute and interrupt the teacher to ask questions. This might have prevented students from asking a lot of questions. A study by Nolan et al. on a neuroanatomy course suggests that sessions focusing on student interaction yield better attendance as opposed to traditional didactic lecture-taking methods [27]. A large-scale study took students’ perceptions of 73 teaching courses into consideration with respect to the course design. It was found that feedback from instructors and active discussion were the two most important factors that improved students’ perceptions of a course [28]. Although the live ZS allowed for immediate resolution of doubts, this came at the cost of affecting the teacher’s flow of thought, which might have led to hesitation from the students' side.

Presentation design

Two factors, namely design of the PPT and use of annotations were favored by students for increasing focus and comprehension (Figures 4, 5, 6). Modern educators need to put more effort into the design of PPTs and actively annotate on the slide being displayed to improve student appeal and engagement.

Background music

Angel et al. reported that background music can have significant effects on cognitive performance. Using fast-tempo music and standardized test batteries, Angel et al. demonstrated that background music increased spatial and linguistic processing skills [29]. Evidently, the development of these skills among students is of utmost importance to facilitate their learning. Fassbender et al. established that using background music while delivering lectures to students can result in a significantly higher recall of facts [30]. In a similar vein, the authors wanted to find out how medical students would react to the presence of background music while studying a complex subject like anatomy. Student opinions were mixed, with 23% of students finding the background music as a necessary aid to improve engagement (Figure 7). Further studies are needed to analyze the effects of using different types of music in online medical lectures.

In summary, online dissection sessions need to be designed by incorporating a healthy mix of the flexibility provided by high-quality pre-recorded videos, the interaction provided via live Zoom sessions, and a well-designed PPT with generous use of annotations to give students a wholesome anatomy learning experience. Forthcoming studies need to assess the merits of such a combination. This study has one limitation. Only the inferior extremity was dissected as a part of this study. Teaching other parts, especially those having smaller anatomical structures and needing finer dissection need to be conducted to further explore the student preferences on the various possible modes for teaching anatomical dissections online. 

## Conclusions

Although virtual dissection teaching methods may not be able to completely replace HOCDs, these results indicate that they can provide a strong impetus for learning in the absence of HOCDs. What needs to be kept in mind however is the incorporation of multiple modes within the same session to increase student engagement. The use of pre-recorded videos covering the most important parts of online dissection sessions enables the students to revisit the more complex concepts that they might miss while learning across the screen in the absence of a face-to-face give and take. It also eliminates the distraction and disengagement that arises due to internet bandwidth problems in the live video conferencing sessions. Maintaining a high level of teacher-student interaction by deliberately dividing longer online sessions into smaller subdivisions with frequent question-answer exchanges is pivotal to encourage student participation. Lastly, while the use of superior quality design and aggressive annotation in online presentations attracts students’ attention, the use of background music and its implications needs to be studied in more detail considering several factors such as the volume, tempo, genre, and other musical parameters that could influence student attention.
